# Claudin-7 is essential for the maintenance of colonic stem cell homoeostasis via the modulation of Wnt/Notch signalling

**DOI:** 10.1038/s41419-024-06658-x

**Published:** 2024-04-23

**Authors:** Kun Wang, Yin Liu, Huimin Li, Xiaoqing Liang, Mengdi Hao, Dajin Yuan, Lei Ding

**Affiliations:** 1 Gastrointestinal Oncology Surgery, Beijing Shijitan Hospital, Capital Medical University, Beijing, China; 2 Department of Ultrasound, Beijing Friendship Hospital, Capital Medical University, Beijing, China

**Keywords:** Cell growth, Intestinal stem cells

## Abstract

Intestinal stem cells (ISCs) play a crucial role in the continuous self-renewal and recovery of the intestinal epithelium. In previous studies, we have revealed that the specific absence of Claudin-7 (Cldn-7) in intestinal epithelial cells (IECs) can lead to the development of spontaneous colitis. However, the mechanisms by which Cldn-7 maintains homeostasis in the colonic epithelium remain unclear. Therefore, in the present study, we used IEC- and ISC-specific Cldn-7 knockout mice to investigate the regulatory effects of Cldn-7 on colonic Lgr5^+^ stem cells in the mediation of colonic epithelial injury and repair under physiological and inflammatory conditions. Notably, our findings reveal that Cldn-7 deletion disrupts the self-renewal and differentiation of colonic stem cells alongside the formation of colonic organoids in vitro. Additionally, these Cldn-7 knockout models exhibited heightened susceptibility to experimental colitis, limited epithelial repair and regeneration, and increased differentiation toward the secretory lineage. Mechanistically, we also established that Cldn-7 facilitates the proliferation, differentiation, and organoid formation of Lgr5^+^ stem cells through the maintenance of Wnt and Notch signalling pathways in the colonic epithelium. Overall, our study provides new insights into the maintenance of ISC function and colonic epithelial homoeostasis.

## Introduction

Adult stem cells play a pivotal role in maintaining homoeostasis and facilitating tissue repair through their capacity for self-renewal and differentiation. Notably, the intestinal epithelium possesses an enhanced capacity for self-renewal, occurring approximately every 4–5 days [1]. This rapid turnover is driven by intestinal stem cells (ISCs) located at the base of epithelial crypts [2]. Leucine-rich repeat-containing G-protein-coupled receptor 5 (Lgr5), a target gene in Wnt signalling, serves as a widely recognised marker for gastrointestinal stem cells [3]. Notably, +4 label-retaining cells are located specifically at the +4 position of the crypt, which is marked by the expression of Bmi1, Hopx, mTert, and Lrig1 [1, 4–8].

Stem cells give rise to transit-amplifying cells, which in turn differentiate into various cell lineages, including nutrient-absorbing enterocytes, protective mucus-secreting goblet cells, antimicrobial compound- and growth factor-producing Paneth cells, hormone-secreting enteroendocrine cells, and immune response-mediating chemosensory Tuft cells [9]. REG4/cKit-labelled deep crypt secretory cells in the colon are functionally similar to Paneth cells, providing specific support for Lgr5^+^ colonic stem cells [10, 11]. These cells migrate toward the intestinal lumen, undergo apoptosis, and are shed into the intestinal lumen, resulting in a continuous turnover of epithelial cells [12]. Lineage-tracing experiments have revealed that Lgr5^+^ ISCs can differentiate into all types of intestinal cells, form intact intestinal crypts, and produce organoids in vitro, which continuously maintain intestinal epithelial homoeostasis [3, 13].

The intestinal epithelium also rapidly regenerates in response to acute injury. Once damaged, the intestinal epithelium undergoes epithelial repair, in which ISCs activate, proliferate, and differentiate to restore the epithelium [14–16]. Acute inflammation has been shown to eradicate Lgr5^+^ ISCs in the small intestine and colon; this depletion of stem cells can delay or inhibit the regeneration of the damaged intestinal epithelium [17, 18]. Despite the robust regenerative capacity of ISCs, severe injury can disrupt intestinal epithelial homoeostasis, leading to inflammatory bowel disease (IBD), such as Crohn’s disease or ulcerative colitis (UC) [19, 20].

Maintaining the integrity of the intestinal barrier is crucial for host intestinal homoeostasis, which relies on the normal morphology and function of intestinal epithelial cells (IECs) and the formation of tight junctions [21, 22]. Claudin-7 (Cldn-7), a tight junction protein, is expressed in the apical, lateral, and basal membranes of IECs, with widespread expression in intestinal crypt stem cells [23]. Our previous research highlighted a significant down-regulation of Cldn-7 expression in UC; additionally, Cldn-7 deficiency led to spontaneous colitis, increasing susceptibility to dextran sulphate sodium (DSS)-induced epithelial injury [24]. Moreover, Cldn-7 has been found to contribute to the maintenance of ISCs homoeostasis in the small intestine [25] and is associated with colorectal cancer stem-like properties [26]. Nonetheless, the underlying mechanisms of Cldn-7-dependent homoeostasis remain unclear.

In the present study, we aimed to investigate the regulatory mechanism between Cldn-7 and colonic stem cells, focusing on their roles in intestinal growth, development, and the progression of UC. Specifically, we explored the role of Cldn-7 in colonic homoeostasis and tissue repair by generating ISC-specific Cldn-7 knockout mice.

## Results

### Cldn-7 is essential for maintaining the homoeostasis of Lgr5^+^ ISCs

**Fig. 1:**
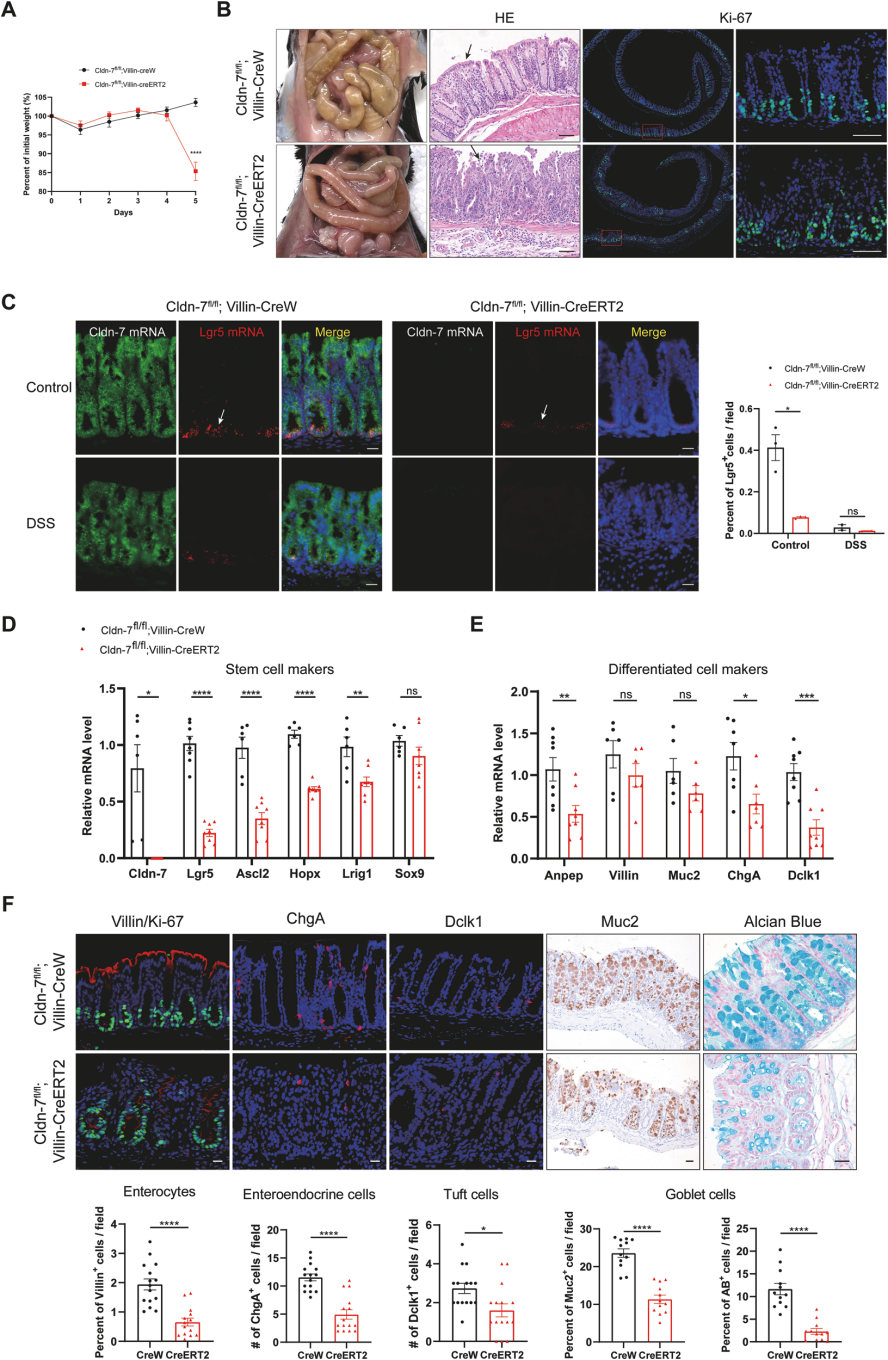
Cldn-7 deletion in intestinal epithelial cells results in the loss of Lgr5^+^ stem cells and impairs differentiation capacity. **A** Body weight curve of mice during TAM induction for 5 days of Cldn-7^fl/fl^;Villin-CreERT2 and Cldn-7^fl/fl^;Villin-CreW mice (*n* = 5 per genotype). **B** Representative images of macroscopic intestine analysis, HE staining, and staining of colonic sections with the proliferation marker Ki-67; scale bars: 50 μm. **C** Representative images of Lgr5 mRNA (red) and Cldn-7 mRNA (green) detected by in situ hybridisation of distal colonic sections from Cldn-7-deficient mice and littermate controls (Left); the white arrow denotes Lgr5-positive cells at the bottom of the colonic crypt. Corresponding statistical analysis of Lgr5-positive cells in the crypt base is shown in the right panel (scale bars: 20 μm; *n* = 3 per genotype). **D**, **E** Quantitative RT-PCR analysis of marker genes for ISCs (Lgr5, Ascl2, Hopx, Lrig1, and Sox9) and differentiated cells (Anpep, Villin, Muc2, ChgA, and Dclk1) in colonic tissues from Cldn-7^fl/fl^;Villin-CreERT2 and Cldn-7^fl/fl^;Villin-CreW mice 5 days after induction (*n* = 6–8 per genotype). **F** Immunohistological staining of colonic sections to detect enterocytes (Villin), enteroendocrine cells (ChgA), goblet cells (Muc2, Alcian blue), and tuft cells (Dclk1); scale bars: 20 μm. For each genotype, the percentage or number of positive cells per field was calculated from 15 fields in 3 mice (Bottom panel). All images are representative of at least three independent experiments. Statistical data are presented as mean ± SEM. All *p* values were calculated using Student’s *t*-test (two-tailed) or Mann-Whitney nonparametric test; *p < 0.05; **p < 0.01; ***p < 0.001; ****p < 0.0001, ns, not significant.

We previously generated IEC-specific Cldn-7 knockout mice (Cldn-7^fl^^/^^fl^;Villin-CreERT2 mice) [27]. The Villin promoter targets Cre expression throughout the intestinal epithelium. Upon Tamoxifen (TAM) administration, Cldn-7 knockout mice exhibited rapid weight loss and intestinal swelling, particularly in the small intestine (Fig. 1A, B). Histopathological staining revealed severe damage to the colon, high infiltration of inflammatory cells, and disruption to the crypt architecture. While most crypts exhibited reduced proliferation, a compensatory increase was observed in a few crypts (Fig. 1B). Given that ISCs are self-renewing cells, crypt destruction in Cldn-7 knockout mice suggests the specific interference of Cldn-7 in the maintenance and function of crypt stem cells.

To further explore this hypothesis, we assessed the expression levels of the ISC-specific marker, Lgr5, in the colonic epithelium. RNAscope results indicated that Lgr5 was localised to the crypt base of the colonic mucosa. IEC-specific Cldn-7 deficiency resulted in the downregulation of Lgr5 expression, with the loss of Lgr5 being more pronounced after DSS administration, highlighting the importance of Cldn-7 in ISC maintenance (Fig. 1C). Additionally, analysis of mRNA levels for various stem cell markers (Lgr5, Ascl2, Hopx, Lrig1, and Sox9) [3, 6, 8, 28, 29] revealed significant downregulation following Cldn-7 knockout (Fig. 1D), supporting the understanding that loss of Cldn-7 induces damage to Lgr5^+^ ISCs.

With Cldn-7 depletion reducing the ISC population, there is an insufficient activation of stem cells to compensate for continued differentiation. Histological staining and quantitative RT-PCR (qRT-PCR) analysis confirmed that Cldn-7 deficiency impaired the differentiation potential of ISCs, affecting the terminal differentiation of enterocytes, goblet cells, enteroendocrine cells, and tuft cells (Fig. 1E, F).

### Cldn-7 null mice exhibit colonic stem cell dysfunction

**Fig. 2:**
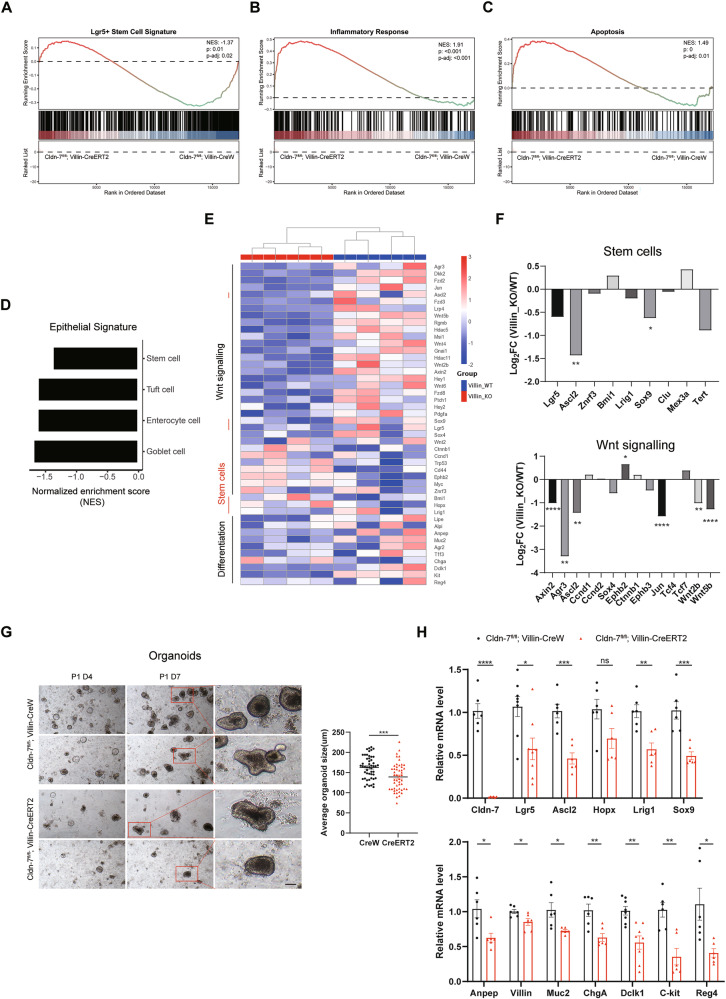
RNA sequencing and organoid culture analysis reveal colonic stem cell dysfunction in Cldn-7 null mice. **A**–**D** Normalised enrichment scores (NES) from GSEA and enrichment plots for intestinal stem cells, differentiated cells, inflammatory responses, and apoptosis transcriptional signatures of Cldn-7^fl/fl^;Villin-CreERT2 and Cldn-7^fl/fl^;Villin-CreW colonic tissues, respectively. **E** Heatmap showing the expression levels of established genes in Wnt signalling pathways, stem cells, and mature differentiated cells in either Cldn-7^fl/fl^;Villin-CreERT2 or Cldn-7^fl/fl^;Villin-CreW colonic tissues. **F** Differential expression analysis of Log_2_ fold change in stem cells and Wnt signalling pathway by Cldn-7 deficiency. **G** Representative images of colonic organoids derived from Cldn-7^fl/fl^;Villin-CreERT2 and Cldn-7^fl/fl^;Villin-CreW mice (Left) from a minimum of three independent experiments; scale bars: 50 μm. The right panel shows the quantification of the average colonic organoid size on day 7 of the first passage. Organoids size was randomly measured in 50 organoids. **H** Quantitative RT-PCR analysis of marker genes for ISCs and differentiated cells in organoids derived from control mice and Cldn-7 knockout mice. (*n* = 3–4 per genotype). All images are representative of at least three independent experiments. Statistical data are presented as mean ± SEM. All *p* values were calculated using Student’s *t*-test (two-tailed) or Mann–Whitney nonparametric test; *p < 0.05; **p < 0.01; ***p < 0.001; ****p < 0.0001, ns, not significant.

To understand the cellular and molecular effects of Cldn-7 deletion on colonic epithelial morphogenesis, we performed RNA-seq analysis on colon tissue extracted from Cldn-7^fl^^/^^fl^;Villin-CreERT2 and Cldn-7^fl^^/^^fl^;Villin-CreW mice. The volcano plot illustrates the 929 upregulated genes and 1378 down-regulated genes identified in the Cldn-7 deletion group (Supplementary Fig. 1A-C). Kyoto Encyclopaedia of Genes and Genomes (KEGG) pathway analysis revealed associations with inflammatory activation, confirming the identified link to severe intestinal inflammation, alongside down-regulation of protein digestion and absorption (Supplementary Fig. 1D). Gene set enrichment analysis (GSEA) identified significant negative enrichment in Lgr5^+^ stem cell-, goblet cell-, enterocyte-, and tuft cell-related genes in Cldn-7 knockout tissues, accompanied by positive enrichment of inflammatory responses and apoptosis (Fig. 2A-D). ISC-related genes, such as Lgr5, Ascl2, Lrig1, Sox9, and Tert, were downregulated, whereas quiescent reserve ISC markers Bmi1 and Mex3a were upregulated. Differentiated cell markers, including Alpi, Anpep, Muc2, Dclk1, Kit, and Reg4, were also significantly downregulated (Fig. 2E, F), aligning with our PCR results.

Next, we analysed pathways closely linked to ISC niche maintenance, proliferation, and differentiation, including the Wnt pathway. Although the Wnt signalling pathway was not significantly enriched, Cldn-7 deletion downregulated the expression of genes downstream of the Wnt signalling pathway, including Axin2, Agr3, Ascl2, Jun, Wnt2b, and Wnt5b. The canonical Wnt target gene and stem cell marker Lgr5 was also significantly downregulated in the intestinal tissue of Cldn-7^fl/fl^;Villin-CreERT2 mice (Fig. 2E, F).

Subsequently, we employed colonic organoid cultures [30] to further investigate the potential of isolated colonic crypts to form clonal multifunctional organoids in the absence of Cldn-7. Compared with Cldn-7^fl^^/^^fl^;Villin-CreW mice, Cldn-7^fl^^/^^fl^;Villin-CreERT2 mice exhibited significantly reduced organoid formation efficiency, organoid diameter, and secondary organoid formation ability (Fig. 2G). qRT-PCR results demonstrated that ISC and differentiation marker genes were significantly downregulated (Fig. 2H), indicating defective organoid formation and differentiation capacities in Cldn-7-deficient crypts.

### Cldn-7 deficiency in ISCs limits Lgr5^+^ ISC expansion during colonic homoeostasis

**Fig. 3:**
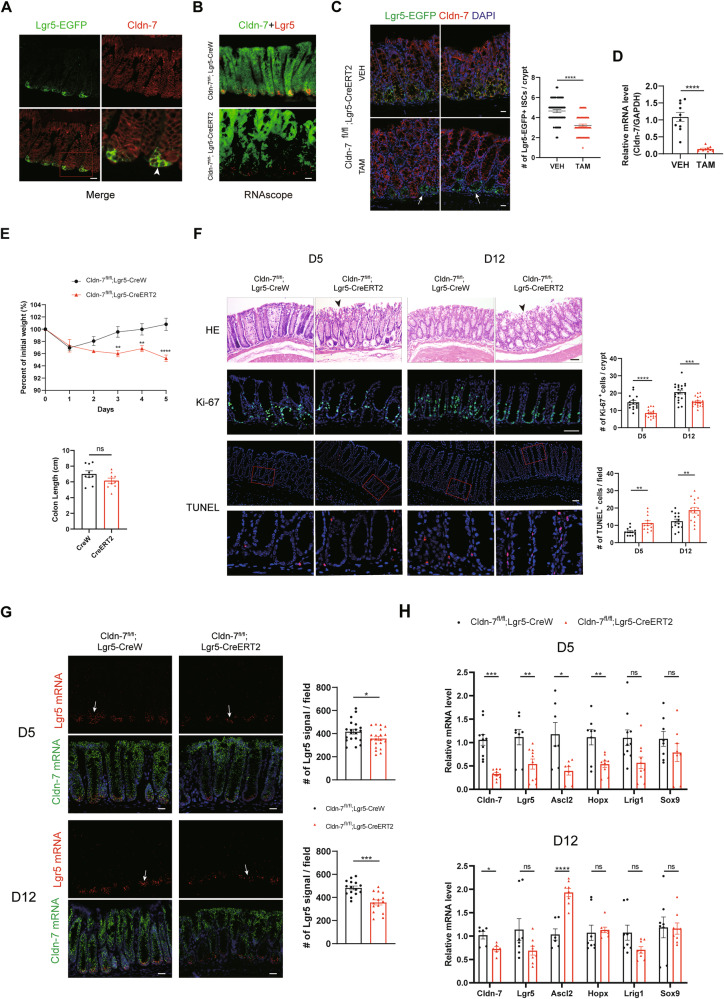
Cldn-7 deficiency reduces Lgr5^+^ ISC numbers and proliferative capacity in vivo. **A** Representative fluorescence images of Lgr5-EGFP and Cldn-7 staining of intestinal crypts. Arrowheads denote Cldn-7 expression (red) in Lgr5^+^ ISCs (EGFP); scale bars: 20 μm. **B** RNAscope analysis confirms the effects of Cldn-7 deletion (green) in the crypt region, detected across a minimum of three independent replicates; scale bars: 20 μm. **C** Immunofluorescence images of distal colonic tissues stained for Cldn-7 (red) and Lgr5-EGFP (green); cell nuclei were counterstained with DAPI (blue). The arrow indicates the absence of Cldn-7 in the crypt area; scale bars: 20 μm. Graphs (right) present the quantification of Lgr5^+^ ISCs in the intestinal crypts (*n* = 3 per genotype). **D** Quantitative RT-PCR analysis of Cldn-7 in sorted Lgr5-EGFP^+^ ISCs by flow cytometry in colonic tissues from Cldn-7^fl/fl^;Lgr5-CreERT2 mice after VEH or TAM administration. (*n* = 5 per genotype). **E** Changes in body weight (*n* = 7 per genotype) and colon length (*n* = 9 per genotype) during TAM induction. **F** Representative images of pathological HE staining, Ki-67 staining, and TUNEL assays of colon sections from Cldn-7^fl/fl^;Lgr5-CreERT2 and Cldn-7^fl/fl^;Lgr5-CreW mice following TAM induction for 5 days (D5) and recovery for 1 week (D12). Arrowheads denote intestinal epithelial disruption. Images are representative of 3 mice per genotype; scale bars: 50 μm. Right panels: quantification of Ki-67-positive cells per crypt base and TUNEL-positive cells per field. **G** RNAscope in situ hybridisation of Lgr5 (red) and Cldn-7 (green) in the colonic mucosa from the two groups of mice during injury and recovery periods; scale bars: 20 μm. The right panels show the quantification of the Lgr5 signal in each field of tissue at different time periods (*n* = 3–4 per genotype). **H** Quantitative RT-PCR analysis of the indicated stem cell markers in colon tissues from Cldn-7^fl/fl^;Lgr5-CreERT2 and Cldn-7^fl/fl^;Lgr5-CreW mice at D5 and D12 (*n* = 4–5 per genotype). Statistical data are presented as mean ± SEM. All *p* values were calculated using Student’s *t*-test (two-tailed) or Mann–Whitney nonparametric test; *p < 0.05; **p < 0.01; ***p < 0.001; ****p < 0.0001, ns, not significant.

To further assess the impact of Cldn-7 deletion on the Lgr5^+^ ISC population, we established ISC conditional Cldn-7 knockout mice (Cldn-7^fl^^/^^fl^;Lgr5-CreERT2) and control mice (Cldn-7^fl^^/^^fl^;Lgr5-CreW). EGFP expression is driven by the endogenous Lgr5 promoter, allowing visualisation of Lgr5^+^ ISCs at the crypt base by detecting EGFP expression [3]. Immunofluorescence results revealed co-expression of EGFP and Cldn-7 in the colonic crypt (Fig. 3A). TAM was administered for 5 days to induce Cldn-7 deletion. RNAscope and immunofluorescence confirmed the significant loss of Cldn-7 expression in the crypt region. Moreover, the number of Lgr5-EGFP^+^ ISCs was significantly reduced after Cldn-7 deletion (Fig. 3B, C). Subsequently, colonic crypts were isolated from TAM-or vehicle control (VEH)-treated Cldn-7^fl/fl^;Lgr5-CreERT2 mice for single-cell flow cytometry, results revealed downregulation of Lgr5-EGFP^+^ ISCs in the TAM treatment group (Supplementary Fig. 2). Cldn-7 expression in sorted EGFP^+^ cells treated with TAM decreased by approximately 85%, confirming the efficient deletion of the Cldn-7 allele in ISCs (Fig. 3D). Moreover, during TAM induction, Cldn-7^fl^^/^^fl^;Lgr5-CreERT2 mice showed aggravated weight loss and shortened colon length (Fig. 3E).

Upon induction of Cldn-7 deficiency, the intestinal epithelial mucosal layer was disrupted, with a slight increase in the number of inflammatory cells present. Next, we performed several assays to determine whether the destruction of colonic epithelial cells and loss of Lgr5^+^ ISCs could be attributed to reduced cell survival. Overall, the number of Ki67^+^ proliferating cells was lower under Cldn-7 deficiency. In Cldn-7^fl^^/^^fl^;Lgr5-CreW colonic mucosa, minimal apoptosis of terminally differentiated epithelial cells was observed on the luminal surface; conversely, Cldn-7^fl^^/^^fl^;Lgr5-CreERT2 mice displayed substantial apoptosis in both the terminal epithelium and the crypt base columnar region. With the continuous renewal of the intestinal epithelium, the proliferation and apoptosis of Cldn-7^fl^^/^^fl^;Lgr5-CreERT2 mice were up-regulated compared with those after TAM induction (Fig. 3F). Meanwhile, in situ hybridisation results revealed strong Lgr5 expression in colonic crypt base cells in the control group, which was significantly reduced after Cldn-7 deletion. Even after one week of recovery, Lgr5 expression remained downregulated compared to that in the Cldn-7^fl^^/^^fl^;Lgr5-CreW group (Fig. 3G). qRT-PCR results confirmed decreased expression of ISCs markers following Cldn-7 deletion. However, mRNA levels of Lgr5, Lrig1, and Sox9 in the colon crypts started to recover at D12 post-TAM induction, whereas Ascl2 and Hopx expression gradually increased, suggesting that the emergence of other reserve stem cell populations may compensate for the initial loss of Cldn7-null ISCs (Fig. 3H).

### Cldn-7 loss in Lgr5^+^ ISCs disrupts differentiation of colonic stem cells

**Fig. 4:**
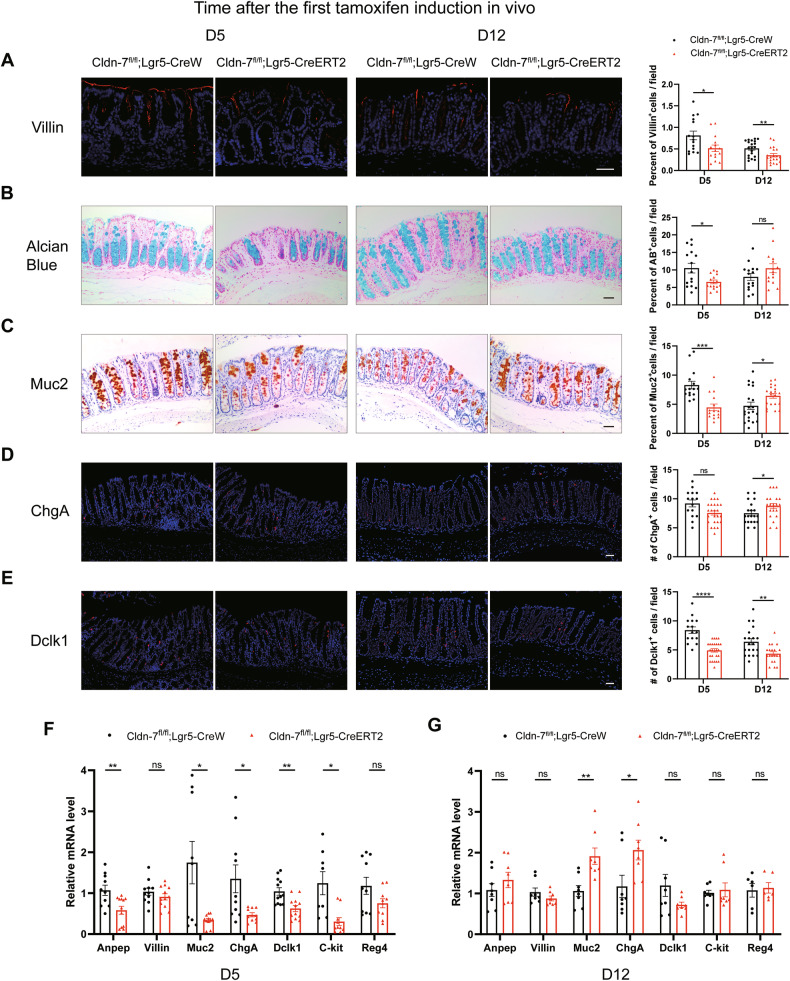
ISC-specific Cldn-7 loss disrupts epithelial differentiation in vivo. Immunohistochemical and immunofluorescence staining were used to detect the abundance of colonic epithelial differentiated cells in Cldn-7^fl/fl^;Lgr5-CreERT2 and Cldn-7^fl/fl^;Lgr5-CreW mice following TAM induction (D5) and 1 week of recovery (D12). **A** Villin marks enterocytes. **B**, **C** Alcian blue and Muc2 mark goblet cells. **D** ChgA marks enteroendocrine cells. **E** Dclk1 marks tuft cells. Nuclei were counter-stained with haematoxylin or DAPI. Images are representative of *n* = 3–4 mice per genotype; scale bars: 50 μm. Quantifications of the number or percentage of positive cells for each differentiated cell are shown beside each representative image. **F**, **G** Quantitative RT-PCR analysis of the indicated differentiated cell markers in colonic tissues from Cldn-7^fl/fl^;Lgr5-CreERT2 and Cldn-7^fl/fl^;Lgr5-CreW mice at D5 and D12 (*n* = 4–5 per genotype). Statistical data are presented as mean ± SEM. All *p* values were calculated using Student’s *t*-test (two-tailed) or Mann–Whitney nonparametric test; *p < 0.05; **p < 0.01; ***p < 0.001; ****p < 0.0001, ns, not significant.

To assess the impact of Cldn-7 depletion on intestinal cell differentiation, we analysed the abundance of mature differentiated cells in the colonic epithelium. Among the intestinal epithelial cells, enterocytes were the most abundant; however, immunofluorescence staining showed that the expression of the enterocyte marker gene was significantly downregulated (Fig. 4A). Localisation analysis of goblet cells using alcian blue staining and immunohistochemical staining for Muc2 revealed a significant reduction after TAM treatment (Fig. 4B, C). Additionally, the expression of enteroendocrine cells and tuft cells was also downregulated (Fig. 4D, E).

Notably, we discovered an interesting phenomenon, an increase in the abundance of secretory lineage cells, such as goblet and enteroendocrine cells, was observed during damage repair of the colonic epithelium in the Cldn-7 deletion group; however, the abundance of absorptive lineage enterocytes remained low in the regenerative colonic epithelium. The results of qRT-PCR showed that the Villin^+^ cells in the Cldn-7^fl/fl^;Lgr5-CreERT2 mice were down-regulated at both the injury and repair period, while the secretory lineage cells (Muc2^+^ goblet cells, ChgA^+^ enteroendocrine cells) were down-regulated at the injury period and significantly increased at the repair stage (Fig. 4F, G).

### Cldn-7 is essential for growth and differentiation of colonic organoids

**Fig. 5:**
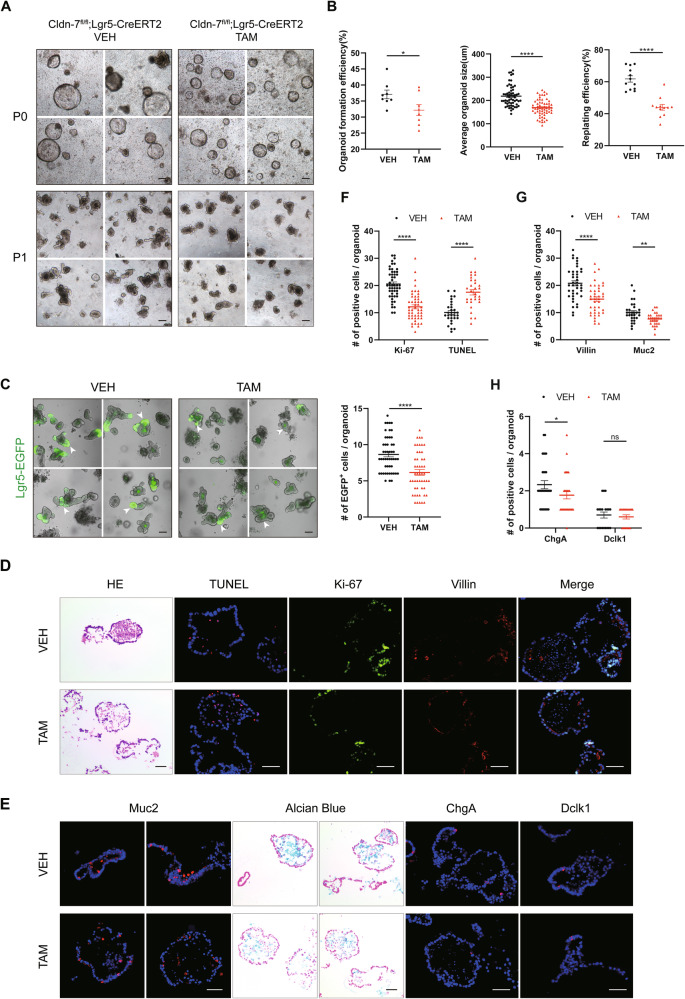
Cldn-7 is necessary for the growth and differentiation of colonic organoids in vitro. **A** Representative bright field images of colonic organoids generated from Cldn-7^fl/fl^;Lgr5-CreERT2 mice treated with VEH or TAM at primary (P0) and first passage (P1); scale bars: 100 μm. **B** Statistical plots of the organoid-forming efficiency of primary cultures, average organoid sizes at day 8 of primary culture, and the replating efficiency at day 5 after first passage. All experiments were repeated three times with at least three mice in each group. **C** Representative image from Lgr5-EGFP^+^ ISCs in VEH- and TAM-treated Cldn-7^fl/fl^;Lgr5-CreERT2 mice, visualised by fluorescent confocal microscopy 5 days after passage. Lgr5-EGFP immunofluorescence is shown in green (white arrowheads); scale bars: 100 μm. The right panel shows the quantification of Lgr5-EGFP^+^ ISCs per organoid. **D**, **E** Representative images of colonic organoids in each group; samples were stained with HE, alongside Ki-67 (green) to detect proliferation, TUNEL (red) to detect apoptosis, Villin (red) to detect enterocytes, Muc2 (red) and Alcian blue to detect goblet cells, ChgA (red) to detect enteroendocrine cells, and Dclk1 (red) to detect tuft cells. Blue represents DAPI staining of nuclei; scale bars: 50 μm. Images are representative of at least three mice. **F**–**H** Statistical quantification of Ki-67, TUNEL, Villin, Muc2, ChgA, Dclk1-positive cells per organoid. Statistical data are presented as mean ± SEM. All *p* values were calculated using Student’s *t*-test (two-tailed) or Mann–Whitney nonparametric test; *p < 0.05; **p < 0.01; ***p < 0.001; ****p < 0.0001, ns, not significant.

To further analyse the functional relevance of Cldn-7 and ISCs, we treated Cldn-7^fl^^/^^fl^;Lgr5-CreERT2 mice with TAM or VEH; then, colonic crypts derived from both groups were used to generate colonic organoids via in vitro culture. Quantitative analysis revealed that TAM-treated organoids exhibited slower growth, reduced diameter, and a significantly reduced organoid formation efficiency. The growth kinetics of the organoids after passage were also significantly different, with the Cldn-7 deletion group displaying significantly downregulated amplification efficiency and reduced formation of crypts (Fig. 5A, B).

Next, we assessed the number of Lgr5-EGFP^+^ ISCs using confocal microscopy. Organoids receiving TAM therapy had fewer crypt structures and exhibited a weak green fluorescence signal; therefore, Cldn-7 deletion can lead to a reduction in Lgr5-EGFP^+^ ISCs (Fig. 5C). Meanwhile, the mRNA expression levels of stem cell-associated genes decreased at the organoid level, further confirming that the stemness of Cldn-7-deficient colonic organoids was inhibited (Supplementary Fig. 3A).

Immunohistochemical examination and qRT-PCR analysis revealed that these organoids replicated the in vivo composite phenotype, with reduced proliferation and increased apoptosis of organoids following Cldn-7 deletion (Fig. 5D, F). The expression of intestinal differentiation markers for enterocytes (Villin), goblet cells (Muc2) and enteroendocrine cells (ChgA) were downregulated (Fig. 5D, E, G, H). qRT-PCR revealed reduced transcription of differentiated cell marker genes in Cldn-7 deficient colonic organoids (Supplementary Fig. 3B).

### Cldn-7 mediates susceptibility to experimental colitis and contributes to intestinal epithelial regeneration

**Fig. 6:**
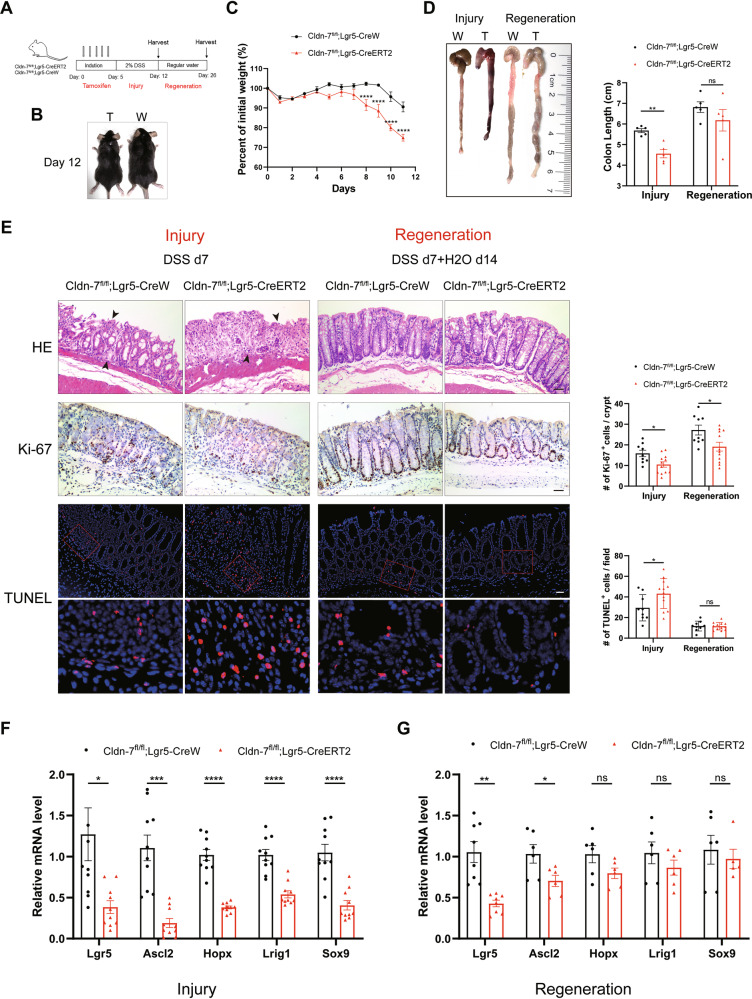
Cldn-7 affects susceptibility to experimental colitis and contributes to intestinal epithelial regeneration. **A** Schematic diagram showing the injury and regeneration phases of experimental colitis in adult Cldn-7^fl/fl^;Lgr5-CreERT2 and Cldn-7^fl/fl^;Lgr5-CreW mice after TAM induction for 5 days. **B** Changes in body size of mice in each group after 3% DSS exposure. **C** Weight loss curves of Cldn-7^fl/fl^;Lgr5-CreERT2 and Cldn-7^fl/fl^;Lgr5-CreW mice during the 5 days of TAM induction and 7 days of DSS treatment (*n* = 5 per genotype). **D** Representative images of colon length changes in mice following DSS induction (Injury) and 2 weeks of recovery (Regeneration). The right panel indicates the corresponding statistical data for colon length analysis (*n* = 5 per genotype). **E** Representative histological HE (top), Ki-67 (middle), and TUNEL (bottom) staining images of colonic tissues from Cldn-7^fl/fl^;Lgr5-CreERT2 and Cldn-7^fl/fl^;Lgr5-CreW mice during acute inflammatory injury (7 days of DSS treatment) and regeneration (7 days of DSS followed by 14 days of regular water consumption). Arrowheads denote extensive mucosal defects and crypt destruction; scale bars: 50 μm. The right panels show the quantification of Ki-67-positive and TUNEL-positive cells in injured and regenerating epithelial tissue (three slides per sample; *n* = 3–4 per genotype). **F**, **G** Quantitative RT-PCR analysis of ISC-related marker genes in intact colonic tissues during intestinal inflammatory injury and regeneration, respectively, in each group of mice. All images are representative of at least three independent experiments. Statistical data are presented as mean ± SEM. All *p* values were calculated using Student’s *t*-test (two-tailed) or Mann–Whitney nonparametric test. *p < 0.05; **p < 0.01; ***p < 0.001; ****p < 0.0001, ns, not significant.

As Cldn-7 depletion resulted in a reduced self-renewal capacity of ISCs, we investigated the role of Cldn-7 in intestinal inflammation and damage repair. To induce colonic epithelial ulceration and inflammation, Cldn-7^fl^^/^^fl^;Lgr5-CreERT2 and Cldn-7^fl^^/^^fl^;Lgr5-CreW mice were treated with 2%DSS for 7 days to induce acute colitis, followed by normal drinking water for 14 days to observe the repair of intestinal injury. After 4 days of treatment, DSS-exposed Cldn-7^fl/fl^;Lgr5-CreERT2 mice showed more pronounced weight loss and displayed clinical symptoms of hypoactivity and lethargy (Fig. 6A–C).

Macroscopic examination of the dissected colon after DSS administration revealed significantly shortened colon lengths and the presence of dark red bloody stools in the intestinal lumen, indicating more severe inflammation in Cldn-7 knockout mice than in control mice (Fig. 6D). Moreover, the colons of Cldn-7 deficient mice displayed damage to the colonic mucosa, complete absence of crypt structures, and infiltration of a large number of immune cells; proliferating cells were scarce in the intestinal epithelium, and apoptosis was significantly upregulated in all layers of the colonic epithelium. In contrast, the control group exhibited a small number of residual crypts and increased cell proliferation/apoptosis ratio (Fig. 6E). qRT-PCR results indicated a significant decrease in ISC markers in the Cldn-7 deletion group following DSS treatment, indicating that Cldn-7 deletion further impaired ISC maintenance (Fig. 6F).

To further understand the impact of Cldn-7 on intestinal epithelial regeneration, we collected colonic tissue 14 days after DSS injury. In both groups, the intestinal structure underwent continuous remodelling. With the expansion of crypts, the cells exhibited compensatory proliferation extending beyond the crypts into the lower 2/3 of the intestinal epithelium; additionally, apoptosis levels were significantly reduced. However, intestinal recovery was slower in the Cldn-7 knockout group than in the control group, with a lower proliferation level and continued down-regulation of ISC-related factors (Fig. 6E, G).

### Increased secretory lineage differentiation in DSS-induced Cldn-7-deficient colonic epithelium

**Fig. 7:**
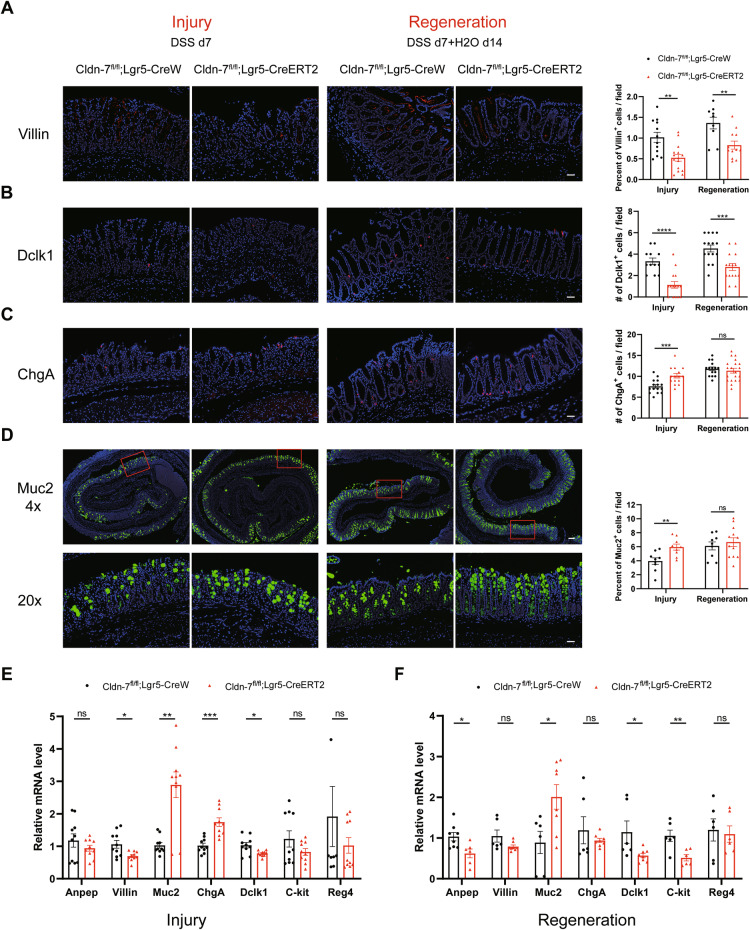
Increased secretory lineage differentiation by DSS-induced Cldn-7-deficient colonic epithelial cells. Immunofluorescence staining was used to detect the expression of colonic epithelial differentiated cells in Cldn-7^fl/fl^;Lgr5-CreERT2 and Cldn-7^fl/fl^;Lgr5-CreW mice during acute injury (DSS d7) and regeneration (DSS d7 + H_2_O d14). **A**–**D** Representative images of (**A**) Villin staining (enterocytes), (**B**) Dclk1 staining (tuft cells), (**C**) ChgA staining (enteroendocrine cells), and (**D**) Muc2 staining (goblet cells), with the lower panels showing the local magnified view of Muc2 staining within the boxes. Nuclei were counter-stained with DAPI; scale bars: 50 μm. Quantifications of the number or percentage of each differentiated cell type during the injury and regeneration period are shown in the right panels (*n* = 3-4 per genotype). **E**, **F** Quantitative RT-PCR analysis of the indicated differentiated cell markers in colon tissues from Cldn-7^fl/fl^;Lgr5-CreERT2 and Cldn-7^fl/fl^;Lgr5-CreW mice during injury and regeneration, respectively (*n* = 35 per genotype). All images are representative of at least three independent experiments. Statistical data are presented as mean ± SEM. All *p* values were calculated using Student’s *t*-test (two-tailed) or Mann–Whitney nonparametric test; *p < 0.05; **p < 0.01; ***p < 0.001; ****p < 0.0001, ns, not significant.

Next, we explored the effects of Cldn-7 depletion on epithelial differentiation during acute inflammation and wound repair. Consistent with the loss of epithelial differentiation following acute Cldn-7 loss, the differentiation ability of intestinal epithelial cells was also impaired under inflammatory conditions. Nonetheless, increased secretory lineage differentiation was observed in the Cldn-7 depletion group during enteritis induction. Specifically, the numbers of enterocytes and tuft cells significantly decreased (Fig. 7A, B), while the numbers of enteroendocrine and goblet cells increased (Fig. 7C, D). Moreover, the colonic epithelium of Cldn-7 knockout mice exhibited sustained high abundances of secretory cell lineages during tissue injury and repair. qRT-PCR results also confirmed these findings that the secretory cell lineage was upregulated and the absorptive lineage was downregulated (Fig. 7E, F).

### Cldn-7 promotes colonic epithelial homoeostasis and regeneration through Wnt and Notch signalling

**Fig. 8:**
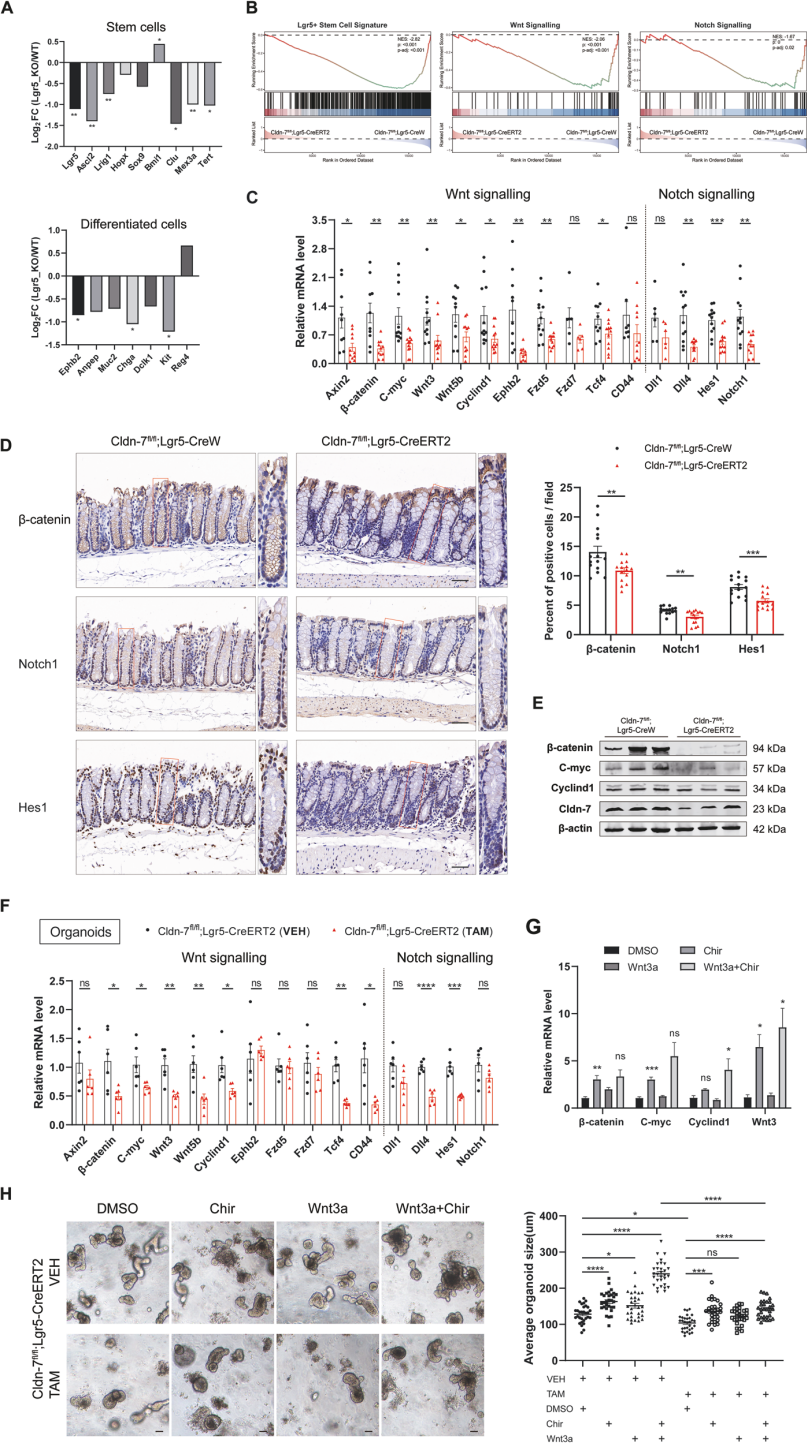
Cldn-7 promotes colonic epithelial homoeostasis and regeneration through the Wnt and Notch signalling pathway. **A** Differential expression analysis showing the Log_2_ fold change values of stem cells and differentiated cells. **B** Representative GSEA enrichment plots showing the negatively enriched gene sets for Lgr5^+^ stem cells, Wnt signalling, and Notch signalling in Cldn-7^fl/fl^;Lgr5-CreERT2 mice. **C** Quantitative RT-PCR verification of Wnt signalling related genes (Axin2, β-catenin, C-myc, Wnt3, Wnt5b, Cyclind1, Ephb2, Fzd5, Fzd7, Tcf4, and CD44) and Notch signalling related genes (Dll1, Dll4, Hes1, and Notch1) in colonic tissues from Cldn-7^fl/fl^;Lgr5-CreERT2 and Cldn-7^fl/fl^;Lgr5-CreW mice treated with TAM for 5 days. *n* = 4–6 per genotype. **D** Immunostains and quantitative analysis of β-catenin, Notch1 and Hes1 labelling in colonic tissues from Cldn-7^fl/fl^;Lgr5-CreERT2 and Cldn-7^fl/fl^;Lgr5-CreW mice treated with TAM for 5 days, scale bars: 50 μm, *n* = 3 per genotype. **E** Immunoblot analysis showing the expression of specific proteins (Cldn-7, β-catenin, C-myc, and Cyclind1) in the colon from Cldn-7^fl/fl^;Lgr5-CreERT2 and Cldn-7^fl/fl^;Lgr5-CreW mice treated with TAM for 5 days. β-actin was employed as a loading control; *n* = 3 per genotype. Data are representative of three independent experiments. **F** Quantitative RT-PCR verification of Wnt and Notch signalling-related genes in colonic organoids derived from Cldn-7^fl/fl^;Lgr5-CreERT2 mice that were treated with VEH or TAM (*n* = 3 per genotype). **G** Colonic organoids derived from Cldn-7^fl/fl^;Lgr5-CreERT2 crypts (VEH or TAM treatment) that were treated with DMSO, Chir (10 μM), Wnt3a (100 ng/mL), or Wnt3a+Chir for 48 h. Quantitative RT-PCR analysis was used to detect β-catenin, C-myc, Cyclind1, and Wnt3 expression in these organoids (*n* = 3 per genotype). **H** Representative images of colonic organoids derived from Cldn-7^fl/fl^;Lgr5-CreERT2 crypts (VEH or TAM treatment) were treated with DMSO, Chir (10 μM), Wnt3a (100 ng/mL), or Wnt3a+Chir; scale bars: 50 μm. Right panel: Quantification of average organoid size per organoid for Cldn-7^fl/fl^;Lgr5-CreERT2 with different treatments. Statistical data are presented as mean ± SEM. All *p* values were generated by Student’s *t*-test (two-tailed) or Mann–Whitney nonparametric test; *p < 0.05; **p < 0.01; ***p < 0.001; ****p < 0.0001, ns, not significant.

To gain a comprehensive understanding of the underlying mechanisms by which Cldn-7 maintains colonic homoeostasis, we conducted sequencing analysis of the colonic epithelium from Cldn-7^fl^^/^^fl^;Lgr5-CreERT2 and Cldn-7^fl^^/^^fl^;Lgr5-CreW mice. RNA-seq results identified 702 differentially expressed genes (DEGs) (padj ≤ 0.05, log_2_ fold change ≥ 1.0), among which 291 were upregulated and 411 were downregulated; notably, the number of DEGs was significantly lower in IEC-specific Cldn-7 knockout mice (Supplementary Fig. 4A, B). The ISC signature genes and differentiated cell lineage genes were significantly downregulated in ISC-specific Cldn-7 knockout mice (Fig. 8A). To analyse the transcriptional changes associated with Cldn-7 loss in ISCs, we performed KEGG and GSEA to compare our RNA-seq dataset with previously established signature gene sets. Lgr5^+^ stem cell gene signalling [4], Wnt signalling [31], and Notch signalling [32] were significantly enriched in downregulated genomes (Fig. 8B, Supplementary Fig. 4C). This simultaneous inhibition of both Wnt and Notch signalling further supported the increased propensity for differentiation into secretory cell lineages [9, 33], consistent with our histological findings.

We further examined the mRNA expression levels of Wnt and Notch signalling pathway-related factors, most of which were downregulated following Cldn-7 deletion (Fig. 8C). Additionally, protein expression levels of key Wnt signalling proteins, including β-catenin, C-myc, and Cyclind1, as well as the Notch receptor Notch1 and Notch target Hes1 were significantly reduced (Fig. 8D, E). Similar results were observed in colitis-induced tissues, where transcription levels and protein levels of Wnt and Notch signalling pathway-related factors were significantly reduced after Cldn-7 depletion (Supplementary Fig. 5A–C). This confirmed that Cldn-7 deletion inhibited transcriptional regulation of the Wnt/Notch signalling pathway, leading to a reduction in stem cells and an increased propensity for differentiation into secretory cells.

To further determine the role of Cldn-7 in signalling regulation, we cultured colonic crypts treated with TAM or VEH to produce colonic organoids. Cldn-7 deficiency was found to inhibit Wnt and Notch signalling at the organoid level, with significant down-regulation of Wnt pathway target genes and Notch pathway-related factors (Fig. 8F). Treatment of cultured organoids with exogenous Wnt3a or GSK3β inhibitor CHIR99021 (Chir), known to promote ISC function [34, 35], revealed that Wnt pathway-related genes were up-regulated in Cldn-7-deficient organoids after Wnt-activating factor treatment (Fig. 8G). The combined Wnt3a and Chir treatment significantly increased the branching of VEH-treated colonic organoids and promoted growth. However, combined treatment with Wnt3a and Chir did not promote TAM-treated colonic organoids to the same extent (Fig. 8H). Overall, this confirms that stimulation of β-catenin via exogenous addition of Wnt3a or Chir can partially rescue the growth defect phenotype of Cldn-7 knockout organoids; nonetheless, Cldn-7 is essential for ISC maintenance and Wnt signalling activation.

## Discussion

The intestinal mucosa relies on continuous epithelial cell renewal and regeneration, driven by the ISC niche, to maintain homoeostasis; consequently, the dysregulation of ISC self-renewal and differentiation has been linked to various intestinal diseases [9, 36]. When intact, the intestinal epithelial barrier can resist infection by bacteria and intestinal pathogens, thereby maintaining a stable microenvironment [37]. However, upon intestinal epithelial injury, colonic stem cell damage exacerbates colitis development. The resulting negative feedback from the inflammatory cascade hinders the survival and regeneration of colonic stem cells, perpetuating a vicious cycle that further compromises intestinal barrier integrity, contributing to IBD development and restriction of regenerative recovery [38].

Our previous research has demonstrated that Cldn-7 expression is reduced in human IBD samples and experimental colitis tissues in mice [24], with the loss of Cldn-7 inhibiting the maintenance of small ISCs and multilineage differentiation [25]. However, the relationship between Cldn-7 and colonic stem cells in colon homoeostasis and inflammation remains unclear. In the present study, we reveal the indispensable role of Cldn-7 in maintaining colonic epithelial homoeostasis via the promotion of ISC proliferation and activity. Notably, we established that ISC-specific Cldn-7 deficiency impairs stem cell stemness, disrupts self-renewal and differentiation, increases susceptibility to DSS-induced colitis, inhibits Wnt and Notch signalling pathways, and promotes secretory lineage differentiation. Overall, this evidence highlights the important role of Cldn-7 in regulating cell fate determination, intestinal epithelial homoeostasis, and inflammatory injury repair.

Specifically, our study revealed that loss of Cldn-7 in the intestinal epithelium leads to ISC damage and compromised differentiation. To further verify the regulatory impact of Cldn-7 on colonic stem cells, we assessed the impact of Cldn-7 deficiency on adult mouse colonic stem cells. Our findings confirmed a gradual reduction in the number of Lgr5-EGFP^+^ ISCs in colonic crypts following Cldn-7 depletion. Active Lgr5^+^ ISCs, located at the base of intestinal crypts, are responsible for regenerating the entire epithelial lineage under normal conditions and, thereby, play a critical role in maintaining intestinal homoeostasis [2, 39]. The ISC niche exhibits high plasticity, with quiescent “+4 ISCs” rapidly regenerating after injury [16, 40]. Our results demonstrated that Cldn-7 deficiency inhibits the stemness of active Lgr5^+^ stem cells and maybe activate the proliferation of Bmi1^+^ reserve stem cells. Meanwhile, acute depletion of Cldn-7 was found to inhibit differentiation into the absorption and secretion lineages, noticeably weakening the stemness capacity of ISCs. DSS-induced experimental colitis has been determined to share pathological features with human UC, including disruption of the intestinal epithelial barrier [41]. In the present study, an experimental colitis model was established by administering DSS through drinking water; this enabled us to explore the role and mechanisms of ISC-specific Cldn-7 deficiency in colitis and colonic epithelial regeneration. Cldn-7 deletion was found to not only intensify intestinal injury but also delay the regeneration of intestinal epithelium injury. Ultimately, these observations strongly indicate that Cldn-7 plays a vital role in the maintenance of intestinal homoeostasis.

Notably, this study determined that the Cldn-7 deletion group exhibited enhanced secretory cell lineage differentiation during colonic epithelial injury repair. This marks the first insight into the role of Cldn-7 in the cell fate decisions of ISCs during injury repair. The balance between stem cell homoeostasis and cell fate decisions in the intestine is tightly regulated by the complex interplay between the Wnt and Notch pathways [42, 43]. The Wnt pathway is considered the dominant force driving Lgr5^+^ ISC proliferation, with the inhibition or loss of Wnt signalling leading to abnormal self-renewal of Lgr5^+^ stem cells or depletion of the stem cell pool, thereby altering lineage development [44, 45]. Meanwhile, the Notch signalling pathway regulates intestinal epithelial fate, inducing ISC differentiation toward the secretory or absorptive lineage; notably, its inhibition initiates stem cell differentiation toward the secretory lineage [46].

Given the significant roles of these cascades in intestinal development and disease, we analysed transcriptomic changes in the colonic epithelium. Our findings indicated that Cldn-7 deletion markedly inhibited both Wnt and Notch signalling pathways; qRT-PCR analysis further validated that Cldn-7 deficiency significantly inhibited the mRNA expression of Wnt- and Notch-related genes, both in vitro and in vivo. This dual inhibition of Wnt and Notch signalling can partly explain the observed reduction in Lgr5^+^ ISC populations and concurrent increase in secretory cell lineages during damage repair. Nonetheless, Wnt signalling activation rescued the growth of Cldn-7-deficient colonic organoids to some extent. These results suggest that Cldn-7 engages in a complex regulatory network with Wnt and Notch pathways, influencing the self-renewal and differentiation of ISCs, while simultaneously maintaining colonic epithelial homoeostasis.

The Wnt signalling pathway possesses a bidirectional regulatory role in maintaining intestinal homoeostasis. Activation of Wnt signalling promotes intestinal homoeostasis via the regulation of Lgr5^+^ ISC self-renewal. However, hyperactivated Wnt signalling has been associated with accelerated proliferation of undifferentiated progenitor cells, leading to intestinal epithelial dysplasia and the onset of colorectal cancer [1, 45, 47]. Therefore, appropriate activation of Wnt signalling is necessary. Our prior study confirmed that the IEC-specific knockout of Cldn-7 promotes Wnt signalling pathway activation in inflammation-associated tumorigenesis [24]. Conversely, In the present study, we highlight the critical role of Cldn-7 deficiency in inhibiting Wnt signalling during normal intestinal regeneration. This indicates that the impact of Cldn-7 on Wnt signalling during normal intestinal epithelial renewal differs from that during tumourigenesis, aligning with previous research. Therefore, it will be very interesting and necessary to further explore the specific mechanism of Cldn-7 regulating Wnt signalling, which is an unavoidable limitation and deficiency. Meanwhile, the specific mechanism of Cldn-7 regulating the Notch signalling pathway is not fully understood and needs to be further studied in the future.

In summary, our study reveals a novel role for Cldn-7 in the regulation of colonic stem cell biology. Specifically, we confirm that Cldn-7 promotes the self-renewal and differentiation of ISCs to maintain colonic environmental homoeostasis via the regulation of Wnt and Notch signalling pathways. Moreover, Cldn-7 deficiency was found to exacerbate intestinal inflammation, delay regeneration of injured epithelium, and promote cell differentiation into the secretory lineage. Overall, these findings provide new insights into the pathogenesis of UC.

## Materials and methods

### Mice

The Cldn-7^fl/fl^;Villin-CreERT2 mice used in this study have been previously described [48]. Cldn-7^fl/fl^;Lgr5-CreERT2 mice were generated through crossbreeding of Lgr5-EGFP-IRES-CreERT2 mice with Cldn-7 flox/flox mice. Lgr5-EGFP-IRES-CreERT2 mice were obtained from the Model Animal Research Centre of Nanjing University as a gift from the Institute of Biophysics, Chinese Academy of Science. For the induction of Cldn-7 deletion, 8–10 week-old Cldn-7^fl/fl^;Lgr5-CreERT2 mice were intraperitoneally injected with 100 μL TAM (20 mg/mL) in sunflower oil for 5 days; sunflower oil (VEH) served as a control. To mitigate the potential off-target effects of TAM and for experiments not assessing GFP expression in ISCs, TAM-exposed Cldn-7^fl/fl^;Lgr5-CreW littermates were used as controls for Cldn-7^fl/fl^;Lgr5-CreERT2 mice. A minimum of three mice for each genotype were used in each experiment. All mice were housed in specific pathogen-free conditions and provided with sterile drinking water. All procedures involving animals adhered to the ARRIVE guidelines and received approval from the Animal Ethics Committee of Beijing Shijitan Hospital Institutional Review Board (sjtkyll-1x-2021(105)).

### Experimental colitis and regeneration

A mouse model of colitis was established according to previously described methods [24]. Briefly, DSS (MP Biomedical, 36,000–50,000 MW) was dissolved in autoclaved water (3%). The resulting solution was sterilised and supplied to the mice for 7 days. To monitor epithelial regeneration, DSS was replaced with normal drinking water for 14 days. Daily body weight measurements were recorded throughout. Finally, the mice were euthanised, and their colons were isolated and washed in ice-cold PBS to eliminate faecal residues; intestinal tissue samples were collected for histological analysis, immunofluorescence, RNA isolation, and protein isolation.

### Intestinal crypt isolation

Mouse intestinal crypts were isolated as previously described with minor modifications [49]. Briefly, colonic crypt isolation and organoid culture were conducted using the IntestiCult system (STEMCELL Technologies), according to the manufacturer’s instructions. Colon tissues were opened longitudinally and washed thoroughly with ice-cold Dulbecco’s phosphate-buffered saline (DPBS) (Thermo Fisher Scientific) containing 1× penicillin/streptomycin and 1× gentamicin (Thermo Fisher Scientific) to remove intestinal contents. The tissues were then decomposed into 5 mm fragments and washed with DPBS until the supernatant was clear. Subsequently, the tissue blocks were transferred to HBSS buffer with 8 mM EDTA and 1 mM DTT and incubated for 40 min at 4 °C on a rocking platform before being vigorously shaken in 10 mL DPBS buffer with 0.1% BSA to release the crypts. The crypt-containing suspension was then filtered through a 70 μm cell strainer (BD Biosciences). Colonic crypts were concentrated via centrifugation at 290×*g* for 5 min, followed by resuspension in 10 mL DMEM/F12 medium (Invitrogen) and centrifugation at 200 ×*g* for 3 min. Finally, we evaluated crypt fractions under an inverted microscope, which enabled the collection of fractions with minimal debris and high crypt yield.

### Organoid culture

Selected crypt fractions were resuspended in a 1:1 mixture of cold Matrigel (Corning) and cold IntestiCult organoid growth medium (STEMCELL Technologies). Subsequently, 50 μL of the crypt suspension was plated at the centre of a preheated 24-well plate, with 500 crypts per well. Following Matrigel polymerisation, 500 μL of prewarmed IntestiCult organoid growth medium supplemented with Primocin (Invivogen) was added. For the first 2 days of culture, 10 μM Y-27632 (ROCK inhibitor; MCE) was added to improve the survival and cloning efficiency of isolated stem cells; the complete medium was changed every 2 days. Upon reaching the appropriate size, organoids were passaged, the medium was removed, and the organoids embedded in Matrigel were digested using Gentle Cell Dissociation Reagent (STEMCELL Technologies). After collection by centrifugation, the organoids were embedded in fresh Matrigel. For Wnt activation treatment, the culture media was supplemented with 10 µM Chir (MCE), 100 ng/mL Wnt3a (PeproTech), or 10 µM Chir + 100 ng/mL Wnt3a; an equal volume of DMSO solution (Sigma-Aldrich) was added to the culture media as a control. The growth state was observed 48 h after the initiation of culture.

### Histology, immunohistochemistry, and immunofluorescence

Intestinal tissues were collected for paraffin embedding, sectioning, and staining, according to standard protocols. Mature organoids were fixed in 4% paraformaldehyde and embedded in OCT for the preparation of frozen sections. Morphological analysis was conducted via haematoxylin and eosin (HE) staining. Additionally, immunohistochemical staining was performed using paraffin-embedded intestinal sections. All sections underwent antigen retrieval, elimination of endogenous peroxidase activity, blocking with goat serum, and subsequent overnight incubation with primary antibodies at 4 °C. Following incubation with horseradish peroxide-labelled secondary antibodies, chromogenic detection was performed using 3,3-diaminobenzidine, with counterstaining of nuclei using haematoxylin.

For immunofluorescence staining of representative intestinal tissue sections and organoid samples, AlexaFluor 488/594 goat anti-mouse or anti-rabbit secondary antibodies were used for fluorescence signalling after incubation with primary antibodies; fluorescent nuclei were counterstained with DAPI. The following primary antibodies were used in this study: rabbit anti-Muc2 (Abcam), rabbit anti-Dclk1 (Abcam), rabbit anti-Ki67 (Abcam), mouse anti-Villin (Santa Cruz), mouse anti-ChgA (Santa Cruz), rabbit anti-GFP (Abcam), and mouse anti-Cldn-7 (Thermo Fisher Scientific). Goblet cells were detected in paraffin-embedded tissue and frozen-embedded organoid sections using an Alcian blue stain kit (Thermo Fisher Scientific). A terminal deoxynucleotidyl transferase-mediated deoxyuridine triphosphate nick-end labelling (TUNEL) assay was performed using a one-step TUNEL apoptosis assay kit (Beyotime), according to the manufacturer’s instructions. All sections were imaged using an inverted optical microscope or laser confocal microscope (Nikon).

### Flow cytometry

To assess the proportion of Lgr5-EGFP^+^ cells, we employed flow cytometry. The isolated crypts from Cldn7^fl/fl^;Lgr5-CreERT2 mice treated with TAM or VEH were dissociated into single cells using TrypLE Express (Thermo Fisher Scientific) supplemented with 2000 U/mL DNase I (Sigma-Aldrich) and 10 μM ROCK inhibitor (Y-27632, MCE) at 37 °C for 30 min. The resulting cell solution was filtered through a 40 μm cell filter before being centrifuged at 2000 rpm and 4 °C for 3 min to precipitate singular cells. Single EGFP-positive cells were sorted using a FACSAria II flow cytometer (BD Biosciences), with DAPI being used to identify dead cell populations. Data analysis was performed using FlowJo software (FlowJo).

### In situ hybridisation

Mouse colons were washed with cold PBS, fixed, dehydrated, and embedded in paraffin. Subsequently, 5 μm sections were prepared, and in situ hybridisation was conducted using the RNAscope Multiplex Fluorescent Reagent kit V2 Assay (ACD) following dewaxing and pre-treatment with target repair, according to the manufacturer’s instructions. The following probes were used for in situ hybridisation: Mm-Lgr5, Mm-Cldn-7-C2, 2-plex Positive Control Probe, and 2-plex Negative Control Probe (ACD). The sections were hybridised with target probes for 2 h at 40 °C in a humidified hybridisation oven. This was followed by signal labelling and amplification using Opal 520 and Opal 570 dyes (ACD). After mounting with ProLong Gold Antifade reagent (Invitrogen), the slides were visualised using a confocal laser scanning microscope (Nikon).

### RNA-seq

Total RNA from colon tissue was extracted using TRIzol reagent (Sigma-Aldrich); each group contained four biological replicates. The RNA with verified integrity was converted into cDNA to produce a cDNA library using the NEBNext Ultra RNA Library Prep Kit for Illumina (NEB). High-throughput sequencing was performed using an Illumina NovaSeq 6000 system (Illumina). The reads were mapped to the full mouse reference genome (GRCm38/mm10). Genes exhibiting a log_2_ fold change ≥ 1 and padj ≤ 0.05 were identified as DEGs. For DEG analysis, Gene Ontology and KEGG enrichment analyses were performed using clusterProfiler (3.8.1) software, with padj < 0.05 as the threshold for enrichment significance. Then, GSEA for specific gene sets was performed using the GSEA analysis platform (http://www.broadinstitute.org/gsea/index.jsp); the corresponding gene lists were compared to the selected genes of interest to identify biological significance.

### RNA isolation and qRT-PCR

Total RNA was extracted from frozen colonic tissues or organoids using TRIzol reagent (Sigma-Aldrich). Subsequently, cDNA was synthesised using a reverse transcription kit (TOYOBO), according to the manufacturer’s instructions. qRT-PCR was performed using SYBR Green reagent (Applied Biosystems). The housekeeping gene GAPDH was used as an internal reference, and the relative expression of each gene was calculated using the 2^−^^ΔΔCt^ method. The gene-specific primers used in this study are listed in Supplementary Table 1.

### Western blot analysis

Total protein was extracted from frozen colonic tissues using PIRA lysates supplemented with protease inhibitors; protein quantification was then performed using a BCA kit (Solaibao Life Science). Equal amounts of total protein were separated on 4–12% SDS-PAGE gels before being transferred to nitrocellulose membranes. the membranes were blocked with 5% non-fat dry milk and incubated with primary antibodies overnight at 4 °C. After washing with TBST, the membranes were incubated with secondary antibodies for 2 h at room temperature. Finally, the immunoreactive bands were imaged using the Ll-COR Odyssey Imaging System (LI-COR). Western blotting was repeated at least three times; representative images have been presented in corresponding figures. The antibodies used in western blotting are listed in Supplementary Table 2. All uncropped immunoblots are shown in Supplementary Fig. 6.

### Statistical analyses

All experiments in this study were independently repeated a minimum of three times, with the number of animals in each experiment being specified in the figure legends. Data visualisation and statistical analyses were performed using GraphPad Prism 8.0.2 software. All data are presented as the mean ± standard error of the mean (SEM). Unpaired Student’s *t*-test (two-tailed) or nonparametric Mann–Whitney *U* test was used for comparisons between the two groups. Changes in body weight over time were analysed using repeated-measures ANOVA. Histopathological examination and statistical analyses were carried out by two authors blinded to experimental design. A *p* value < 0.05 was considered statistically significant.

### Supplementary information

Supplementary Materials

## Data Availability

The authors declare that all data supporting the findings of this work are available within the main text and the supplementary information files, or from the corresponding author upon reasonable request.
