# Supplementary material for: Claudin-7 is essential for the maintenance of colonic stem cell homoeostasis via the modulation of Wnt/Notch signalling
Source: Cell Death Dis. 2024 Apr 23;15(4):284. doi: 10.1038/s41419-024-06658-x (PMC11039680; doi:10.1038/s41419-024-06658-x)
Supplement: Supplementary file 1 — Supplementary Materials [file 41419_2024_6658_MOESM1_ESM.pdf]

Supplementary Table 1. Primer sequences used for the qRT-PCR assays.

**Gene**	**Species**	**Forward (5'-3')**	**Reverse (5'-3')**
GAPDH	Mouse	CATGTTTGTGATGGGTGTGAACCA	AGTGATGGCATGGACTGTGGTCAT
Cldn-7	Mouse	ATGATGAGCTGCAAAATGTACG	GCGACAAACATGGCTAAGAAG
Lgr5	Mouse	TTTTTCTGTCAAGTGCTCTTCG	CAGGACACATAGCAAAACGATC
Ascl2	Mouse	CAACAAGAAGCTGAGTAAGGTG	GGAGTAGCGGGTGTTAACAG
Hopx	Mouse	TGGAGTACAACTTCAACAAGGT	CTAGTCCGTAACAGATCTGCAT
Lrig1	Mouse	CGCAGCCTAAACCTGAGTTATA	CATTGCTGTTGAGGTACACTTC
Sox9	Mouse	CGGAACAGACTCACATCTCTCC	GCTTGCACGTCGGTTTTGG
Anpep	Mouse	GCCCATTCCATTTCTCAAAAGT	GTAGCCGGTTACGTTAATGTTC
Villin	Mouse	GATCCCTTCAAGTGGAGTAACA	GACTGGTATTGGCAGTGAAAAC
Muc2	Mouse	CGAGCACATCACCTACCACATCATC	TCCAGAATCCAGCCAGCCAGTC
ChgA	Mouse	CCAAGGTGATGAAGTGCGTC	GGTGTCGCAGGATAGAGAGGA
Dclk1	Mouse	CGAACTCTCTCGGATAATGTGA	TGTACTCCAGCTTCTTAAAGGG
C-kit	Mouse	ACCACTCCTGTCTCACCAGT	GGGCCTGGATTTGCTCTTTGT
Reg4	Mouse	GAGAAACCTGCCTGTGTGGATTGG	GCTTCACTCTTTGTCCTGGGATTCC

Supplementary Table 2. Key resources used in this experiment.

Reagent or resources	Source	Identifier
**Antibodies**		
Mouse anti-Cldn-7	Thermo Fisher	Cat#: 37-4800
Rabbit anti-GFP	Abcam	Cat#: ab183734
Rabbit anti-Muc2	Abcam	Cat#: ab272692
Mouse anti-Villin	Santa Cruz	Cat#: sc-58897
Mouse anti-ChgA	Santa Cruz	Cat#: sc-393941
Rabbit anti-Dclk1	Abcam	Cat#: ab109029
Rabbit anti-Ki-67	Abcam	Cat#: ab15580
Rabbit anti-β-catenin	Abcam	Cat#: ab32572
Rabbit anti-C-myc	Abcam	Cat#: ab32072
Rabbit anti-Cyclind1	Abcam	Cat#: ab134175
Rabbit anti-Notch1	Abcam	Cat#: ab52627
Rabbit anti-Hes1	Abcam	Cat#: ab71559
Mouse anti-β-actin	Abcam	Cat#: ab8226
Alexa Fluor® 790 Donkey anti-rabbit IgG	Abcam	Cat#: ab175780
Alexa Fluor® 680 Goat anti-mouse IgG	Invitrogen	Cat#: A21058
Alexa Fluor® 594 Goat anti-mouse IgG	Abcam	Cat#: ab150116
Alexa Fluor® 488 Goat anti-rabbit IgG	ZSGB-Bio	Cat#: ZF-0511
Alexa Fluor® 594 Goat anti-rabbit IgG	ZSGB-Bio	Cat#: ZF-0516
**Chemicals and recombinant proteins**		
DAPI	Solaibao Life Science	Cat#: C0060
RNAscope Probe-Mm-Lgr5	Advanced Cell Diagnostics	Cat#: 312171

RNAscope Probe-Mm-Cldn-7-C2	Advanced Cell Diagnostics	Cat#: 437941-C2
TRIzol reagent	Sigma-Aldrich	Cat#: T9424
SYBR Green Mix	Applied Biosystems	Cat#: A25742
TrypLE Express	Thermo Fisher	Cat#: 12604013
Matrigel Matrix	Corning	Cat#: 356231
Tamoxifen	Sigma-Aldrich	Cat#: T5648
DSS	MP Biomedicals	Cat#: 216011090
Y27632	MCE	Cat#: HY-10071
DMSO	Sigma-Aldrich	Cat#: D2650
CHIR-99021	MCE	Cat#: HY-10182
Wnt3a	PeproTech	Cat#: 351-20
GCDR reagent	Stem Cell Technologies	Cat#: 100-0485
**Critical commercial assays**		
IntestiCult Organoid Growth Medium	Stem Cell Technologies	Cat#: 06005
RNAscope Multiplex Fluorescent Reagent Kit	Advanced Cell Diagnostics	Cat#: 323100
TUNEL apoptosis assay kit	Beyotime	Cat#: C1089
Alcian Blue Kit	Thermo Fisher	Cat#: G1565
qPCR RT Master Mix	TOYOBO	Cat#: FSQ-101
BCA Protein Assay Kit	Solaibao Life Science	Cat#: PC0020
Immunohistochemistry kit	ZSGB-Bio	Cat#: PV-6001
DAB substrate kit	ZSGB-Bio	Cat#: ZLI-9017

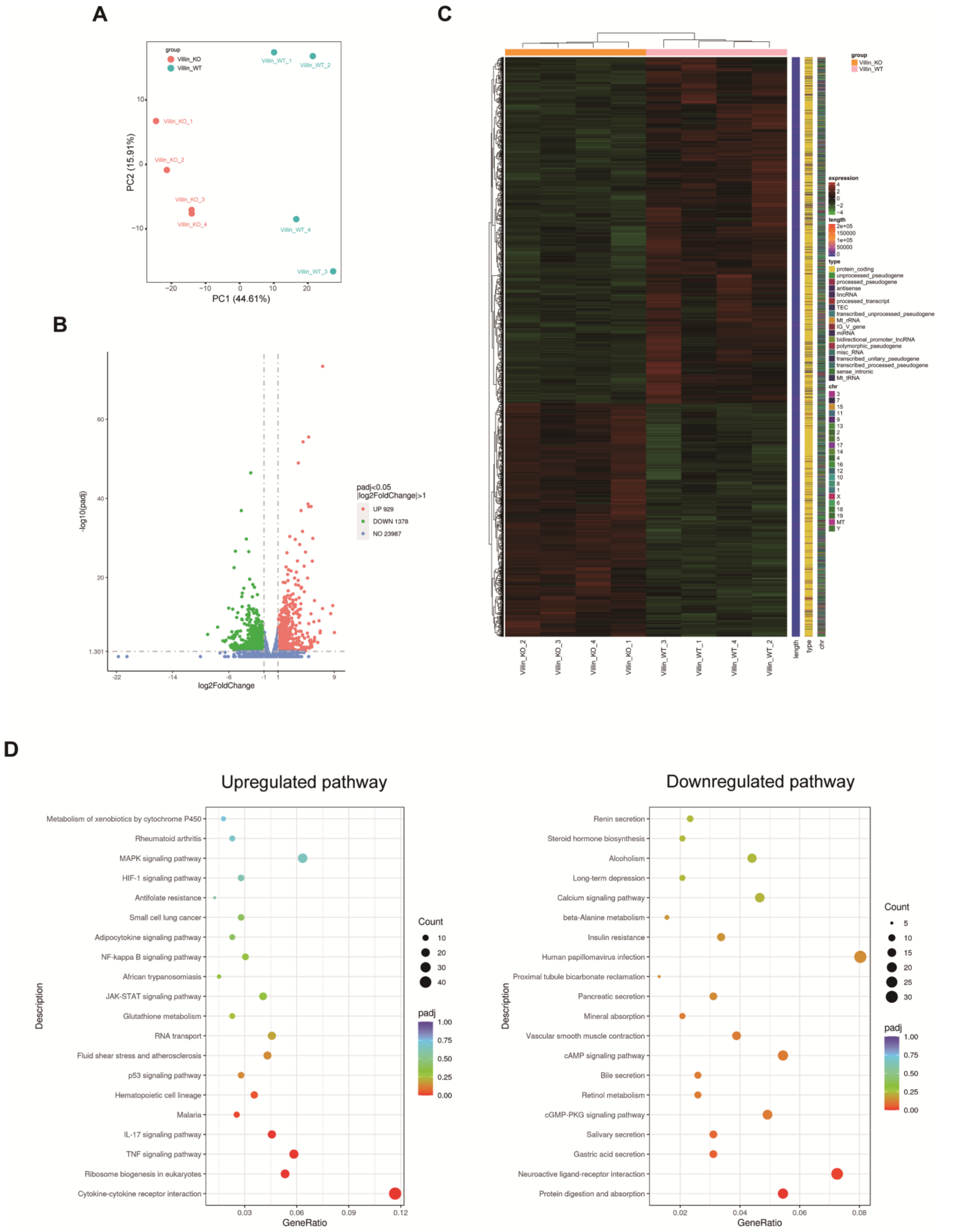

Supplementary Fig. 1 Analysis of transcriptome sequencing data from Cldn-7$^{fl/fl}$;Villin-CreERT2 mice.

A Principal component analysis (PCA) plot of RNA-seq data from Cldn-7$^{fl/fl}$;Villin-CreERT2 and Cldn-7$^{fl/fl}$;Villin-CreW colonic tissues; n=4 per genotype. **B** Volcano plot showing the upregulated and

downregulated differentially expressed genes (DEGs) in colonic tissues from Cldn-7$^{fl/fl}$;Villin-CreERT2 mice compared to those in Cldn-7$^{fl/fl}$;Villin-CreW mice. Genes with padj < 0.05 and $\log_2$ fold change (Log_2 FC) > 1 were identified as DEGs. **C** Hierarchical clustering heatmap of DEGs. **D** KEGG pathway enrichment analysis indicating up- and down-regulated pathways following Cldn-7 deletion.

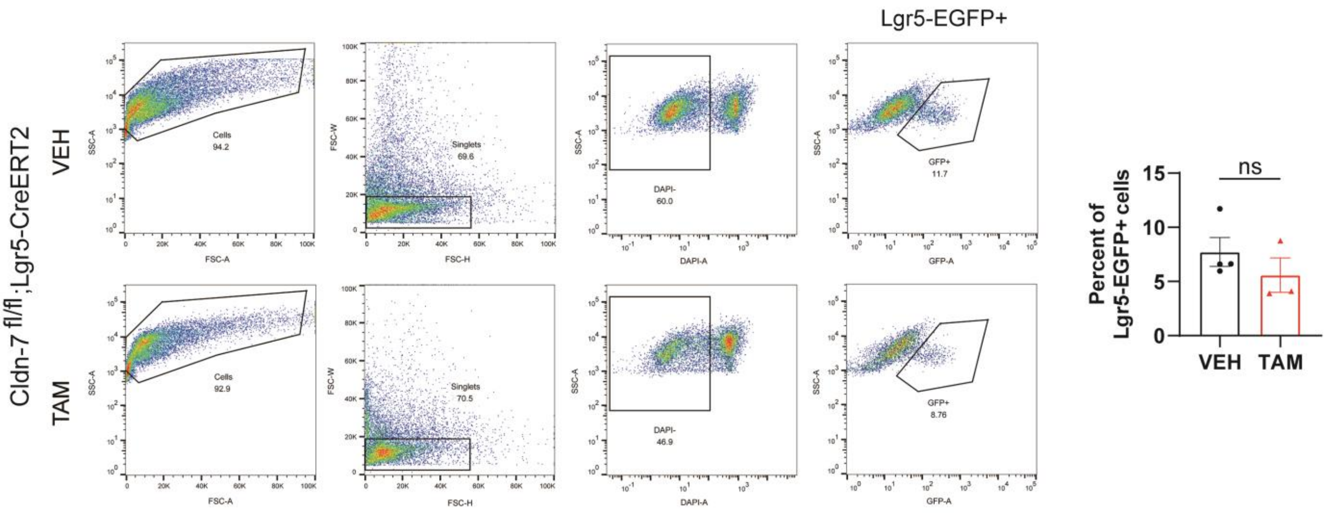

Supplementary Fig. 2 Analysis of flow cytometry data. Flow cytometry analysis of EGFP expression (quantitative marker for Lgr5$^+$ stem cells) in colonic tissues from Cldn-7$^{fl/fl}$;Lgr5-CreERT2 mice after VEH or TAM administration. The right panel shows the quantification of Lgr5-EGFP$^+$ cells in both treatments (n=3-4 per genotype). Statistical data are presented as mean ± SEM. All p values were calculated using Student's t-test (two-tailed) or Mann-Whitney nonparametric test.

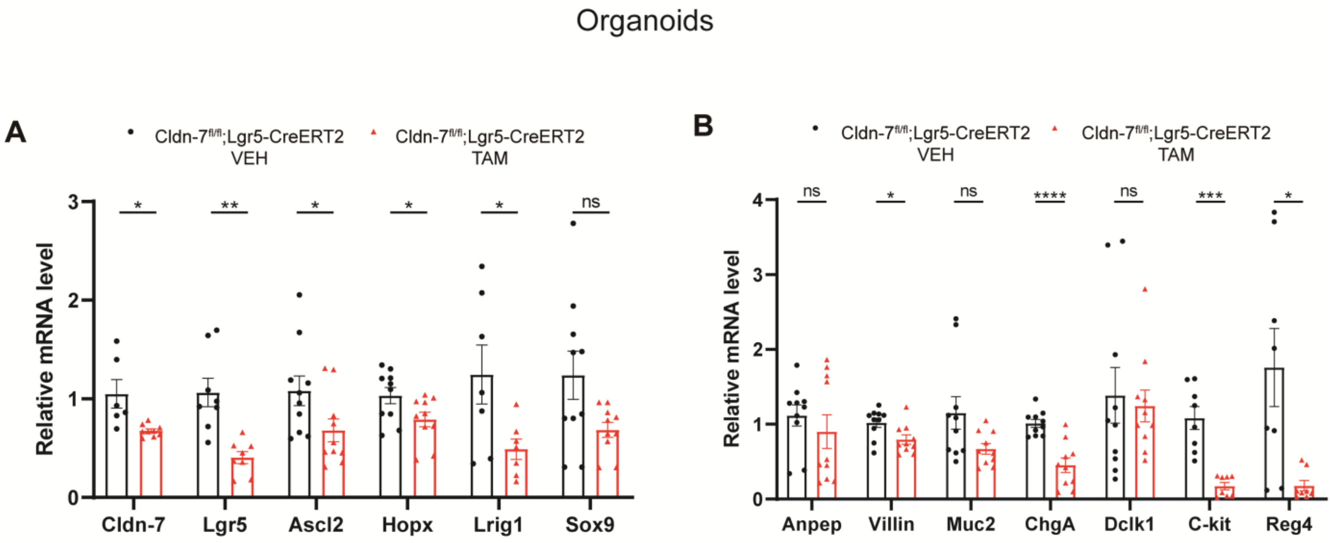

Supplementary Fig. 3 Quantitative RT-PCR analysis of colonic organoid. A Quantitative RT-PCR analysis of ISC-related marker genes in colonic organoids from Cldn-7$^{fl/fl}$;Lgr5-CreERT2 mice following VEH or TAM treatment (n=4-5 per genotype). **B** Quantitative RT-PCR analysis of intestinal differentiated cells-related factors in colonic organoids from Cldn-7$^{fl/fl}$;Lgr5-CreERT2 mice following VEH or TAM treatment (n=4-5 per genotype). Statistical data are presented as mean ± SEM. All p values were calculated using Student's t-test (two-tailed) or Mann-Whitney nonparametric test; *p < 0.05; **p < 0.01; ***p < 0.001; ****p < 0.0001, ns, not significant.

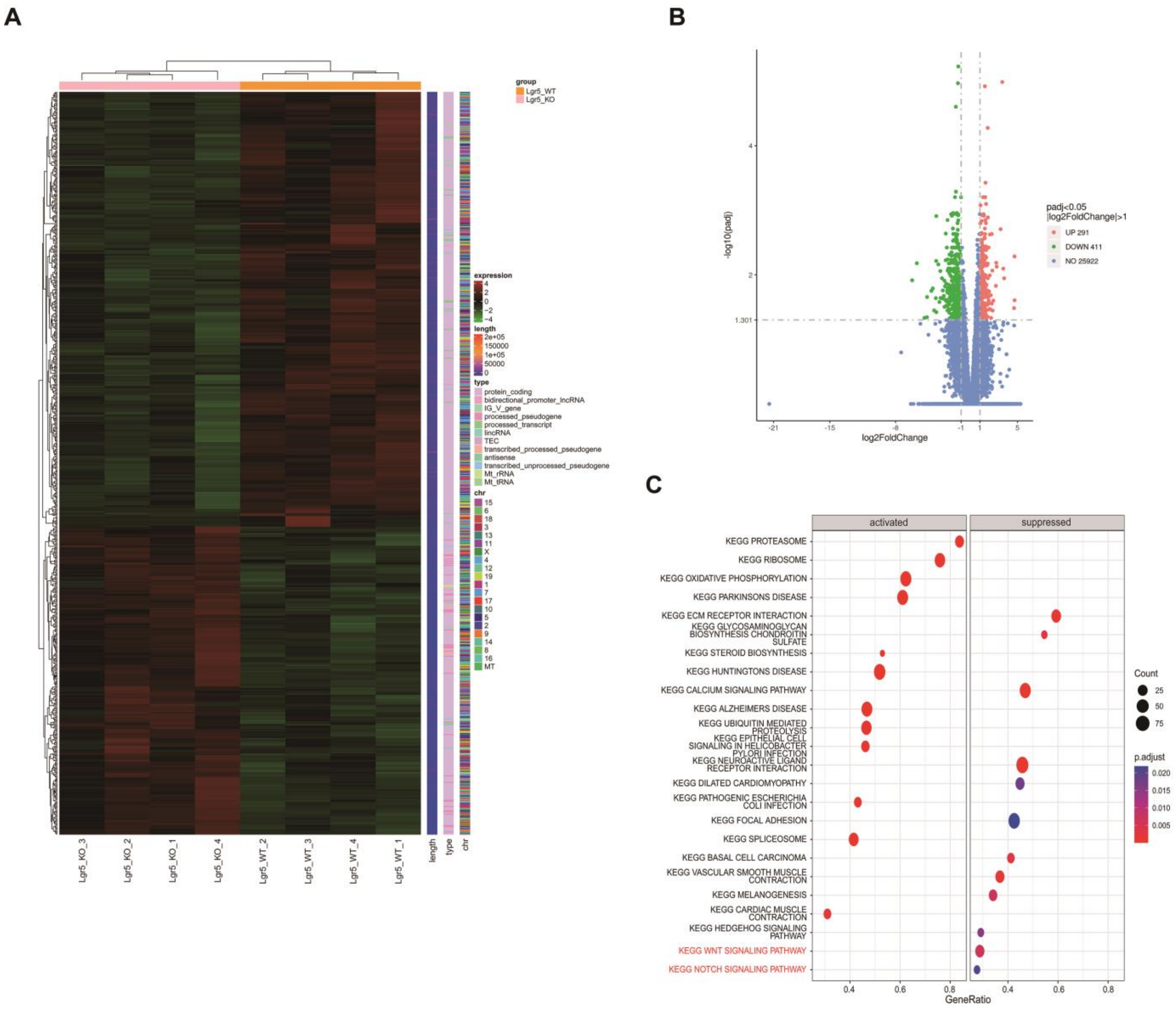

Supplementary Fig. 4 Analysis of transcriptome sequencing data from Cldn-7$^{fl/fl}$;Lgr5-CreERT2 mice.

A RNA-seq analysis of colonic tissues from TAM-treated Cldn-7$^{fl/fl}$;Lgr5-CreERT2 and Cldn-7$^{fl/fl}$;Lgr5-CreW

mice (n=4 per genotype). Differentially expressed genes (padj<0.05 and $\log_2$ fold change > 1) were identified and clustered via a hierarchical clustering heatmap. **B** Volcano plot showing the upregulated and downregulated differentially expressed genes. **C** KEGG pathway enrichment analysis indicating activated and suppressed pathways in Cldn-7$^{fl/fl}$;Lgr5-CreERT2 vs Cldn-7$^{fl/fl}$;Lgr5-CreW mice.

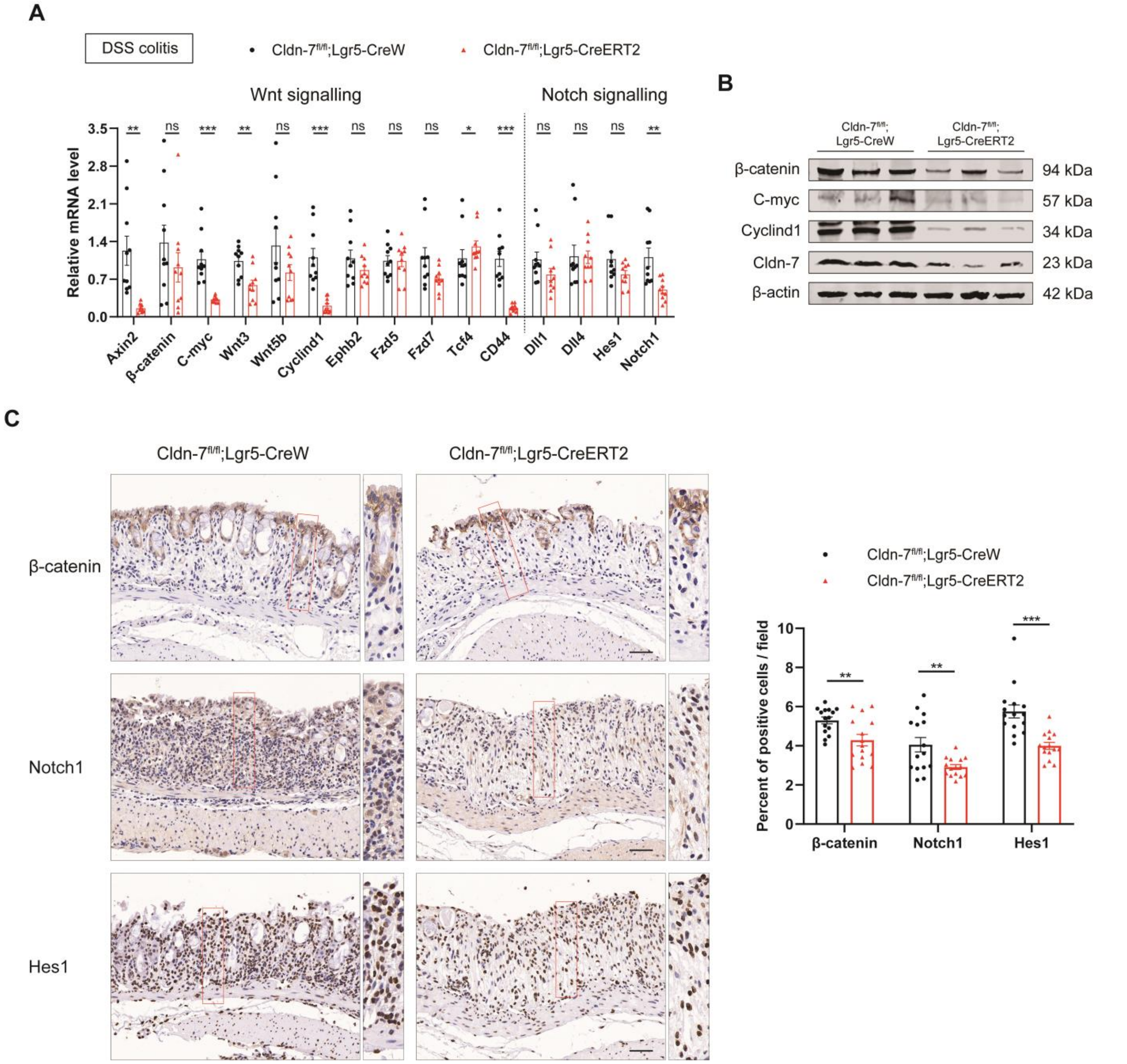

Supplementary Fig. 5 Cldn-7 deficiency inhibits Wnt and Notch signalling in colonic inflammatory epithelium. A Quantitative RT-PCR verification of Wnt signalling related genes (Axin2, β-catenin, C-myc, Wnt3, Wnt5b, Cyclind1, Ephb2, Fzd5, Fzd7, Tcf4, and CD44) and Notch signalling related genes (Dll1, Dll4, Hes1, and Notch1) in colonic tissues from Cldn-7$^{fl/fl}$;Lgr5-CreERT2 and Cldn-7$^{fl/fl}$;Lgr5-CreW mice treated

with DSS for 7 days. n=4-6 per genotype. **B** Immunoblot analysis showing the expression of specific proteins (Cldn-7, β-catenin, C-myc, and Cyclind1) in the colon from Cldn-7$^{fl/fl}$;Lgr5-CreERT2 and Cldn-7$^{fl/fl}$;Lgr5-CreW mice treated with DSS for 7 days. β-actin was employed as a loading control; n=3 per genotype. Data are representative of three independent experiments. **C** Immunostains and quantitative analysis of β-catenin, Notch1 and Hes1 labeling in colonic tissues from Cldn-7$^{fl/fl}$;Lgr5-CreERT2 and Cldn-7$^{fl/fl}$;Lgr5-CreW mice treated with DSS for 7 days, scale bars: 50 μm, n=3 per genotype. Statistical data are presented as mean ± SEM. All p values were calculated using Student's t-test (two-tailed) or Mann-Whitney nonparametric test; *p < 0.05; **p < 0.01; ***p < 0.001; ns, not significant.

Fig.S5B

Fig.8E

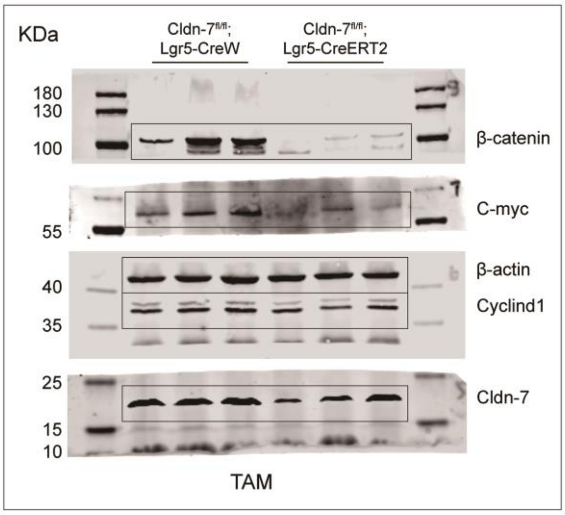

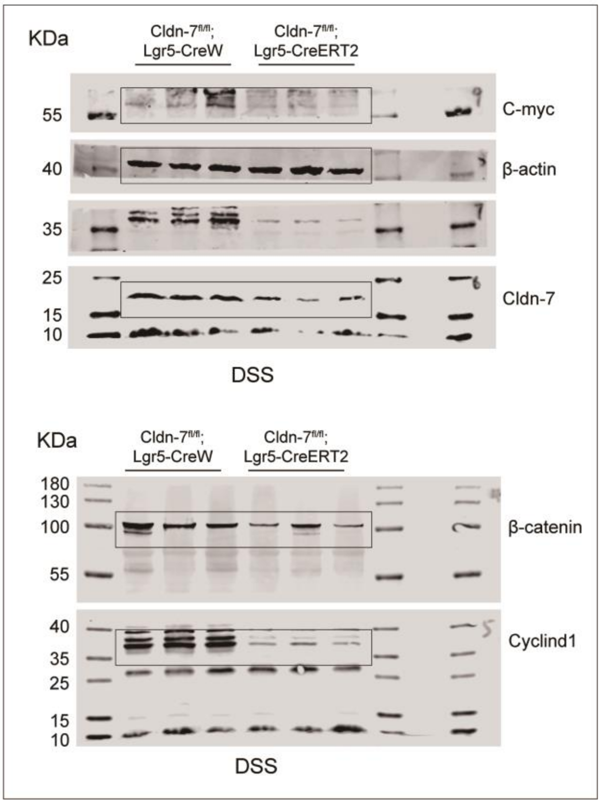

Supplementary Fig.6 Full immunoblots.
